# Isolation and characterization of a novel wheat cysteine-rich receptor-like kinase gene induced by *Rhizoctonia cerealis*

**DOI:** 10.1038/srep03021

**Published:** 2013-10-23

**Authors:** Kun Yang, Wei Rong, Lin Qi, Jiarui Li, Xuening Wei, Zengyan Zhang

**Affiliations:** 1 The National Key Facility for Crop Gene Resources and Genetic Improvement, Key Laboratory of Biology and Genetic Improvement of Triticeae Crops, Ministry of Agriculture, Institute of Crop Science, Chinese Academy of Agricultural Sciences, Beijing 100081, China; 2 Central South University of Forestry and Technology, Changsha, Hunan 412001, China; 3 Department of Plant Pathology, Kansas State University, Manhattan, KS 66502

## Abstract

Cysteine-rich receptor kinases (CRKs) belong to the receptor-like kinase family. Little is known about CRK genes in wheat. We isolated a wheat CRK gene *TaCRK1* from *Rhizoctonia cerealis*-resistant wheat CI12633 based on a differentially expressed sequence identified by RNA-Sequencing (RNA-Seq) analysis. *TaCRK1* was more highly expressed in CI12633 than in susceptible Wenmai 6. Transcription of *TaCRK1* in wheat was induced in CI12633 after *R. cerealis* infection and exogenous abscisic acid (ABA) treatment. The deduced TaCRK1 protein contained a signal peptide, two DUF26 domains, a transmembrane domain, and a serine/threonine protein kinase domain. Transient expression of a green fluorescence protein fused with TaCRK1 in wheat and onion indicated that TaCRK1 may localize to plasma membranes. Characterization of *TaCRK1* silencing induced by virus-mediated method in CI12633 showed that the downregulation of *TaCRK1* transcript did not obviously impair resistance to *R. cerealis*. This study paves the way to further CRK research in wheat.

Plants live in complex environments in which they are continuously threatened by a broad range of pathogens, including viruses, bacteria, fungi, and oomycetes. To defend themselves against pathogen attack, plants have evolved sophisticated strategies to perceive infection and translate this perception into effective immune responses^1^. Receptor-like kinases (RLKs) play fundamental roles in perceiving external stimuli, activating downstream signaling pathways, and regulating cellular behavior in response to pathogen infection. Most plant RLKs are composed of an extracellular domain, a single-stranded transmembrane domain, and a cytoplasmically localized domain with serine/threonine kinase activity^2^. The extracellular domain is thought to function in recognition and binding of a specific ligand, the transmembrane domain to anchor the protein in a membrane, and the protein kinase domain to transduce the signal^2^,^3^. RLKs have been identified in many plant species and have been implicated in regulating the processes of plant growth, development, and responses to biotic and/or abiotic stresses^4^. For instance, two LRR-RLKs, FLAGELLIN SENSTIVE2 (FLS2) and bacterial translation elongation factor EF-Tu receptor (EFR), act as pattern-recognition receptors (PRRs) to detect pathogen-associated molecular patterns (PAMPs) and trigger immune responses in *Arabidopsis thaliana*^5^,^6^,^7^,^8^. The chitin elicitor receptor kinase 1 of *Arabidopsis* (AtCERK1) directly binds chitin through its lysine motif (LysM)–containing ectodomain to activate immune responses^9^,^10^.

Cysteine (C)-rich receptor kinases (CRKs) are a sub-group of plant RLKs, which are characterized by one or more repeats of unknown function 26 (DUF26) domains consisting of a C-X8-C-X2-C motif. The DUF26 domain, also known as stress-antifungal domain (PF01657), has antifungal activity^11^ and played a role in salt stress response^12^. To keep consistence, the DUF26 nomenclature will be used throughout this article. The conserved cysteine residues in these extracellular domains of RLKs may maintain the CRK three-dimensional structure through disulphide bridges and form zinc finger motifs to mediate protein-protein interaction^13^. In *Arabidopsis*, there are more than 40 CRKs, constituting a large subgroup of the RLK family^13^. To date, the function of only a few CRKs in *Arabidopsis thaliana* have been described. Several CRKs from *Arabidopsis* were induced by the phytohormone salicylic acid (SA), pathogen infection, and reactive oxygen species (ROS)^14^,^15^,^16^,^17^,^18^,^19^,^20^,^21^,^22^. Overexpression of *AtCRK5* and *AtCRK13* in transgenic plants conferred increased resistance to the bacterial pathogen *Pseudomonas syringae*^14^,^15^. Over-expression of *AtCRK5* and three close homologs, *AtCRK4*, *AtCRK19*, and *AtCRK20*, driven by a chemically inducible promoter, activated hypersensitive responses including cell death^15^,^16^. Moreover, *PvPK20-1*, a CRK gene isolated from roots of the common bean, is also differentially regulated by pathogens, symbionts, and nodulation factors^23^. These studies suggested that some CRK proteins are involved in plant responses to biotic and/or abiotic stresses.

Wheat (*Triticum aestivum*) is one of the most important staple crops in the world and plays a fundamental role in food security. Sharp eyespot, mainly caused by soil-borne fungal pathogen *Rhizoctonia cerealis*, is one of the most devastating diseases of wheat^24^,^25^. In infected wheat plants, *R. cerealis* may destroy the transport tissues in stems and sheaths of host plants, causing blocked transportation of substances required for nutrition, lodging, and even dead spikes^24^. Previous studies have demonstrated several genes in wheat defense response to *R. cerealis*, such as *TaERF3*^26^ and *TaGluD*^27^; however, very little is known about the roles of RLKs in wheat defense response to *R. cerealis*. To explore whether RLK proteins function in wheat defense response to *R. cerealis*, we used RNA-Sequencing (RNA-Seq) to study transcript patterns of RLK genes in resistant and susceptible wheat genotypes toward *R. cerealis* infection.

In this paper, a novel CRK gene in wheat, *TaCRK1*, was isolated based on a differentially expressed sequence. The expression of *TaCRK1* was markedly induced after infection with *R. cerealis* and by exogenously applied ABA in resistant line CI12633. We investigated the subcellular localization of the TaCRK1 protein and also analyzed the function of *TaCRK1* in wheat defense response to *R. cerealis*.

## Results

### *TaCRK1* was induced by *R. cerealis* infection in resistant line CI12633

**Figure 1:**
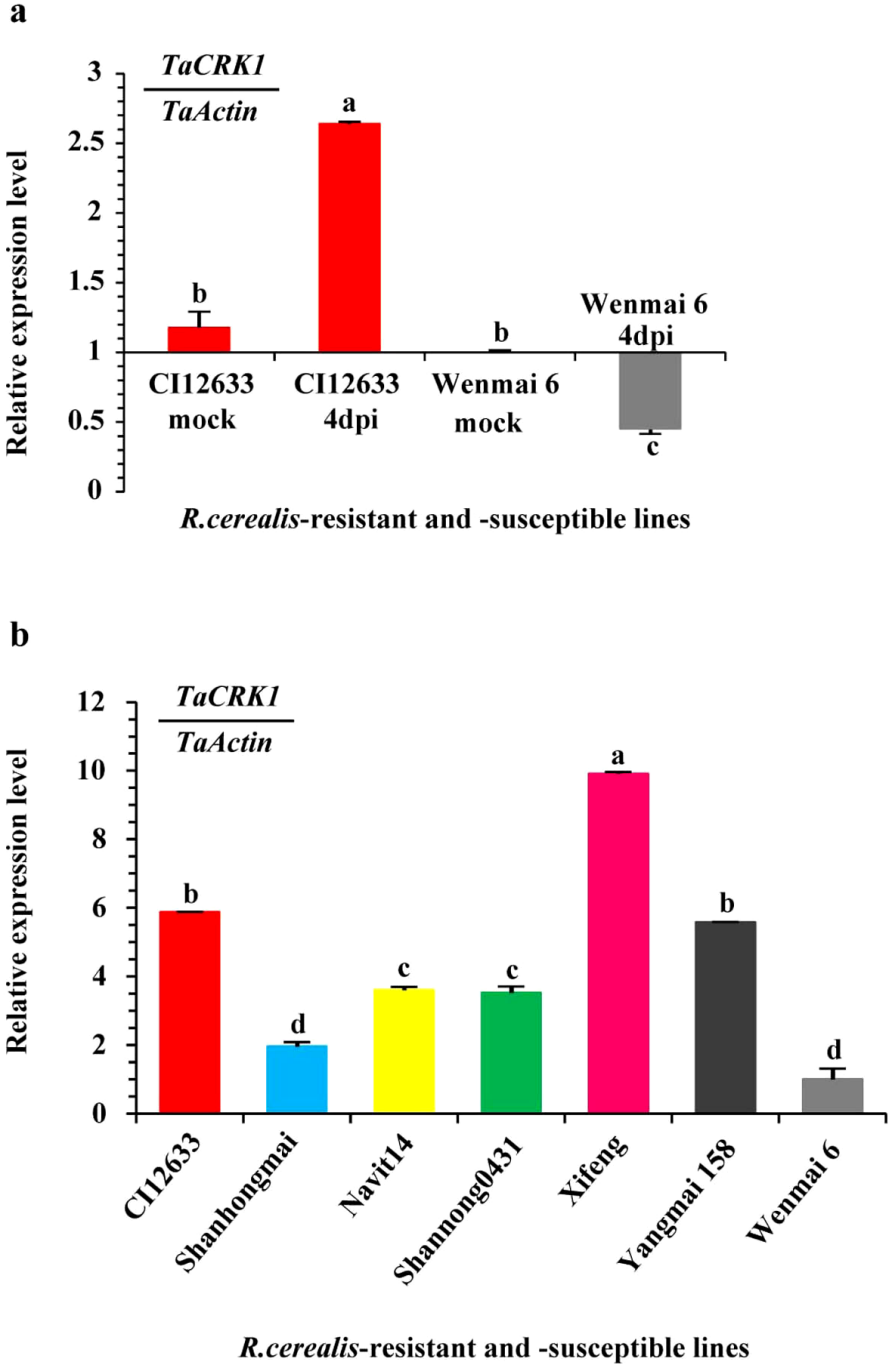
Transcriptional patterns of *TaCRK1* in wheat responding to *Rhizoctonia cerealis* inoculation were measured by qRT-PCR. (a) Transcription of *TaCRK1* in *R. cerealis*-resistant wheat CI12633 and susceptible wheat Wenmai 6 following mock and *R. cerealis* treatments. Relative transcriptional level of *TaCRK1* indicated the changing fold of the gene transcript over that in mock-treated Wenmai 6. (b) Transcriptional patterns of *TaCRK1* in *R. cerealis*-resistant lines and susceptible lines after *R. cerealis* inoculation for 4 days. Transcriptional level of *TaCRK1* in other 6 wheat lines was relative to highly susceptible Wenmai 6. The average and standard error (SE) of three replicates were presented. The transcript abundances with different letters are significantly different from each other based on statistical comparison.

To identify wheat RLK genes in response to *R. cerealis* infection, RNA-Sequencing (RNA-Seq) analysis was used to compare transcriptome differences of *R. cerealis*-resistant line CI12633 and *R. cerealis*-susceptible cultivar Wenmai 6 under *R. cerealis* inoculation. Based on RNA-seq data, gene ontology (GO) analyses, and pathway analyses, wheat cDNA clone AK330939 (GenBank accession no.AK330939) was identified to show two-fold increase in transcriptional level in the *R. cerealis-*inoculated CI12633 relative to the mock-treated CI12633. Moreover, at 4 days post inoculation (dpi) with *R. cerealis*, the transcriptional level of AK330939 was elevated 2.6-fold in the resistant wheat CI12633 compared with the susceptible wheat Wenmai 6. This gene, hereafter designated as *TaCRK1*, showed homologous to the genes encoding cysteine-rich receptor-like protein kinases in plants. The transcriptional levels of *TaCRK1* in CI12633 and Wenmai 6 were further evaluated by real-time quantitative reverse RT-PCR (qRT-PCR). The result of qRT-PCR assay (Fig. 1a) was consistent with the RNA-Seq analysis. As shown in Figure 1a, the transcriptional level of *TaCRK1* was elevated 2.2-fold in *R. cerealis*-inoculated CI12633 relative to mock-treated CI12633, but down-regulated 2.2-fold in *R. cerealis*-inoculated Wenmai 6 compared with mock-treated Wenmai 6. The expression abundance of the gene was significantly higher in CI12633 than in Wenmai 6 at 4 dpi with *R. cerealis*. These results suggested that *TaCRK1* may be involved in wheat defense response to *R. cerealis* infection.

The transcriptional level of the *TaCRK1* gene was also investigated via qRT-PCR analyses in the stems of seven wheat lines/cultivars with different levels of resistance and susceptibility to *R. cerealis* at 4 dpi. The experimental wheat lines/cultivars include resistant lines CI12633 and Shanhongmai; moderate-resistant lines Xifeng, shannong0431, and Navit14; moderate susceptible line Yangmai 158; highly-susceptible line Wenmai 6, whose disease indexes after *R. cerealis* infection were shown in Supplementary Table 1. As shown in Fig. 1b, the transcriptional level of *TaCRK1* was the highest in moderate-resistant line Xifeng and the lowest in highly susceptible cultivar Wenmai 6. However, the transcriptional levels of *TaCRK1* were not consistent with the resistance degrees in other tested wheat lines/cultivars. For example, compared with the susceptible line Yangmai 158, the relative transcriptional level of *TaCRK1* was lower in more resistant Shanhongmai. These results suggested that the expression levels of *TaCRK1* in the seven wheat lines/cultivars at 4 dpi were not associated with their resistance degrees.

### *TaCRK1* encodes a cysteine-rich receptor-like protein kinase

**Figure 2:**
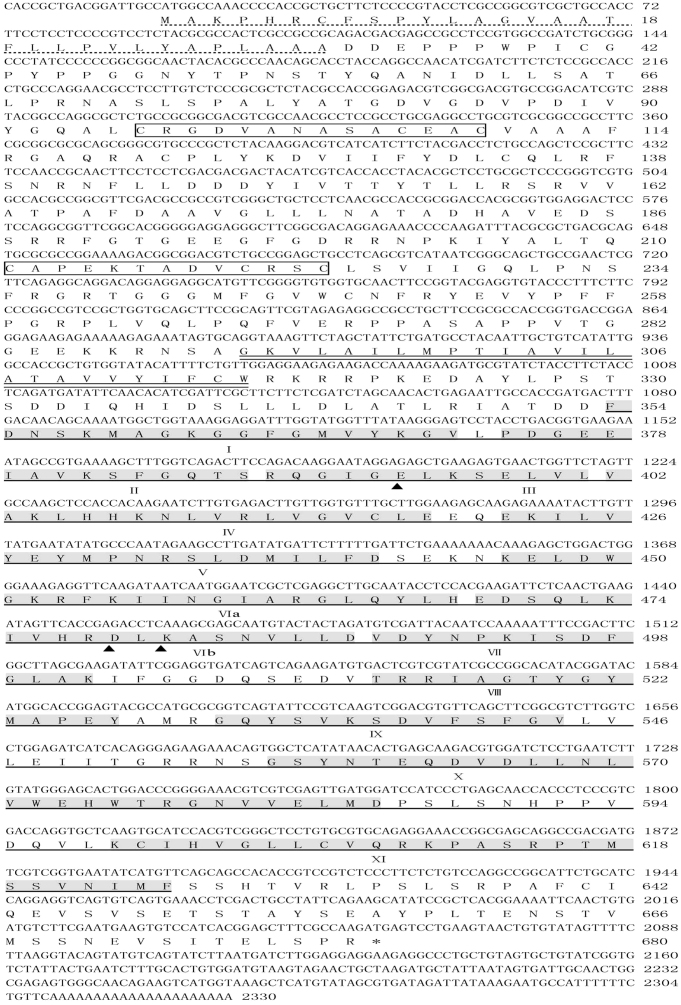
Deduced amino acid sequence of the wheat cysteine-rich repeat receptor-like protein kinase (TaCRK1). The conserved cysteine-rich repeat (CRR) motif is marked by the open box and located between the signal peptide (indicated by dotted line) and the transmembrane domain (represented by double line). The kinase domain (underlined) follows the transmembrane domain. Roman numbers mark the eleven subdomains conserved in the plant serine/threonine protein kinase family. Arrowheads indicate the three kinase catalytic sites.

The 3′ un-translated region (UTR) of *TaCRK1* was cloned by 3′ rapid amplification of cDNA ends (RACE), and the full open reading frame (ORF) sequence was amplified from *R. cerealis*-infected stem cDNA of CI12633. The cDNA sequence of *TaCRK1* with 2330-bp length was obtained through analyzing the overlaid sequences and deposited in the public GenBank database (GenBank accession no. KC818618). Sequence analysis showed that the cDNA of *TaCRK1* includes an ORF consisting of 2043 nucleotides (from 19 to 2061 nucleotides) (Fig. 2). BLAST analysis showed that the nucleotide sequence of this gene was highly similar to those of predicted receptor-like protein kinases from *Brachypodium distachyon* (GenBank accession no. XM_003560070) (83% identity) and rice (GenBank accession no. AK111650) (77% identity). We compared the nucleotide sequence and amino acid sequence of *TaCRK1* with that of AK330939. The nucleotide sequences of *TaCRK1* and AK330939 share 95.7% identity. The deduced amino acid sequence of TaCRK1 shares 96.9% identity with that of AK330939 (Supplementary Fig. 1). The predicted TaCRK1 protein sequence exhibits 18 amino acid substitutions and one insertion compared with the deduced AK330939 protein. Among these, six amino acid substitutions occur in protein kinase catalytic domain. These results suggested that *TaCRK1* and AK330939 were homologous, but not identical.

The deduced TaCRK1 protein contains 680 amino acid residues with a molecular weight of 74.93 kD and a pI of 6.01. The search for protein conserved domain using InterPro-Scan web indicated that the TaCRK1 protein contains a signal-peptide domain, two DUF26 domains, a transmembrane domain, and a serine/threonine protein kinase catalytic domain that includes 11 subdomains (Fig. 2). The predicted result using Smart software was consistent with that from InterPro-Scan.

**Figure 3:**
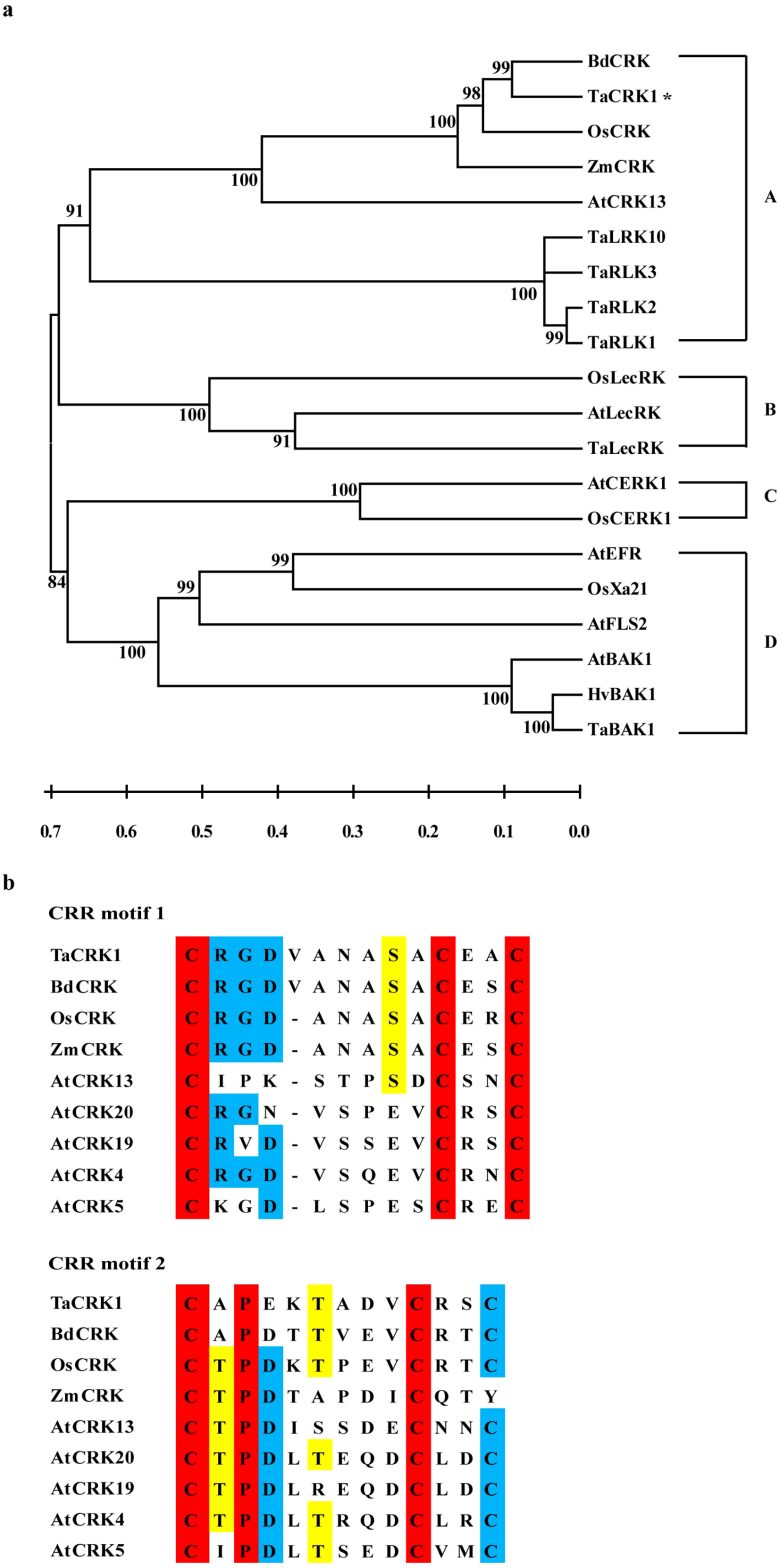
Phylogenetic analysis of the deduced amino acid sequences of TaCRK1 and 19 additional RLKs and comparison of CRR motif sequences. (a) Phylogenetic tree constructed by neighbor-joining algorithms of MEGA 5.0 software after the multiple RLK protein sequences alignment using the CLUSTAL W program. Bootstrapping was performed 1,000 times to obtain support values for each branch. Four groups of RLK proteins, including cysteine-rich RLK (CRK), Lectin RLK (LecRK), LysM RLK (CERK), and LRR RLK, were represented by letters A, B, C and D, respectively. The GenBank accession numbers of RLK protein sequences are as follows: AtCRK13 (AEE84724), ZmCRK (AFW74556), OsCRK (BAC65053), BdCRK (XP_003560118), TaRLK10 (AAC49629), TaRLK3 (AAQ82627), TaRLK2 (ABB84341), TaRLK1 (ABB84340), AtLecRK (AEE79957), OsLecRK (AAT77694), TaLecRK (ACN41357), AtCERK1 (BAF92788), OsCERK1 (D7UPN3), AtBAK1 (AEE86224), HvBAK1 (AEE44134), TaBAK1 (ACD49737), AtEFR (AED92850), AtFLS2 (AED95370), and OsXa21 (AAC80225). (b) Amino acid alignment of CRR motifs between TaCRK1 and CRK proteins. Boxes in red represent 100% similarity, blue for 75% similarity, and yellow for 50% conserved amino acids.

Phylogenetic analysis was performed to decipher the relationship between TaCRK1 and related RLKs in other plant species. Using MEGA 5.0, 20 available RLK sequences from different plant species were constructed to a neighbor-joining phylogenetic tree, which consisted of four different subgroups of RLKs: LRR-RLK, LysM-RLK (CERK), Lectin-RLK (LecRK), and cysteine-rich RLK (CRK). TaCRK1, BdCRK, ZmCRK, OsCRK, and AtCRK13 were clustered into the clade of cysteine-rich RLK (Fig. 3a). Next, we performed a multi-alignment on amino acid sequences of TaCRK1, BdCRK (GenBank accession no. XP_003560118), ZmCRK (GenBank accession no. AFW74556), OsCRK (GenBank accession no. BAC65053), AtCRK20 (GenBank accession no. O65479), AtCRK19 (GenBank accession no. NP_194058), AtCRK4 (GenBank accession no. NP_190172), AtCRK5 (GenBank accession no. NP_567677), and AtCRK13 (GenBank accession no. AEE84724) using DNAMAN software. TaCRK1 is more closely related to the predicted *B. distachyon* CRK BdCRK, which showed the highest sequence identity (83%) to TaCRK1. OsCRK in rice and ZmCRK in maize have 77% and 70% identities with TaCRK1, respectively. The sequence identities between TaCRK1 and *Arabidopsis* CRKs are only 39%–44%. All of the above CRKs in *Brachypodium distachyon*, rice, maize, and *Arabidopsis* contain two extracellular DUF26 domains, in which each DUF26 domain contains one cysteine-rich repeat (CRR) motif (Fig. 3b). Thus, these CRK proteins contain two CRR motifs in their DUF26 domains. The first CRR motifs of CRKs in wheat and *Brachypodium distachyon* contain C-X9-C-X2-C motif, which is distinguished from C-X8-C-X2-C motif of CRKs in rice, maize, and *Arabidopsis*.

### TaCRK1 protein was likely to be localized to the plasma membrane *in* *planta*

**Figure 4:**
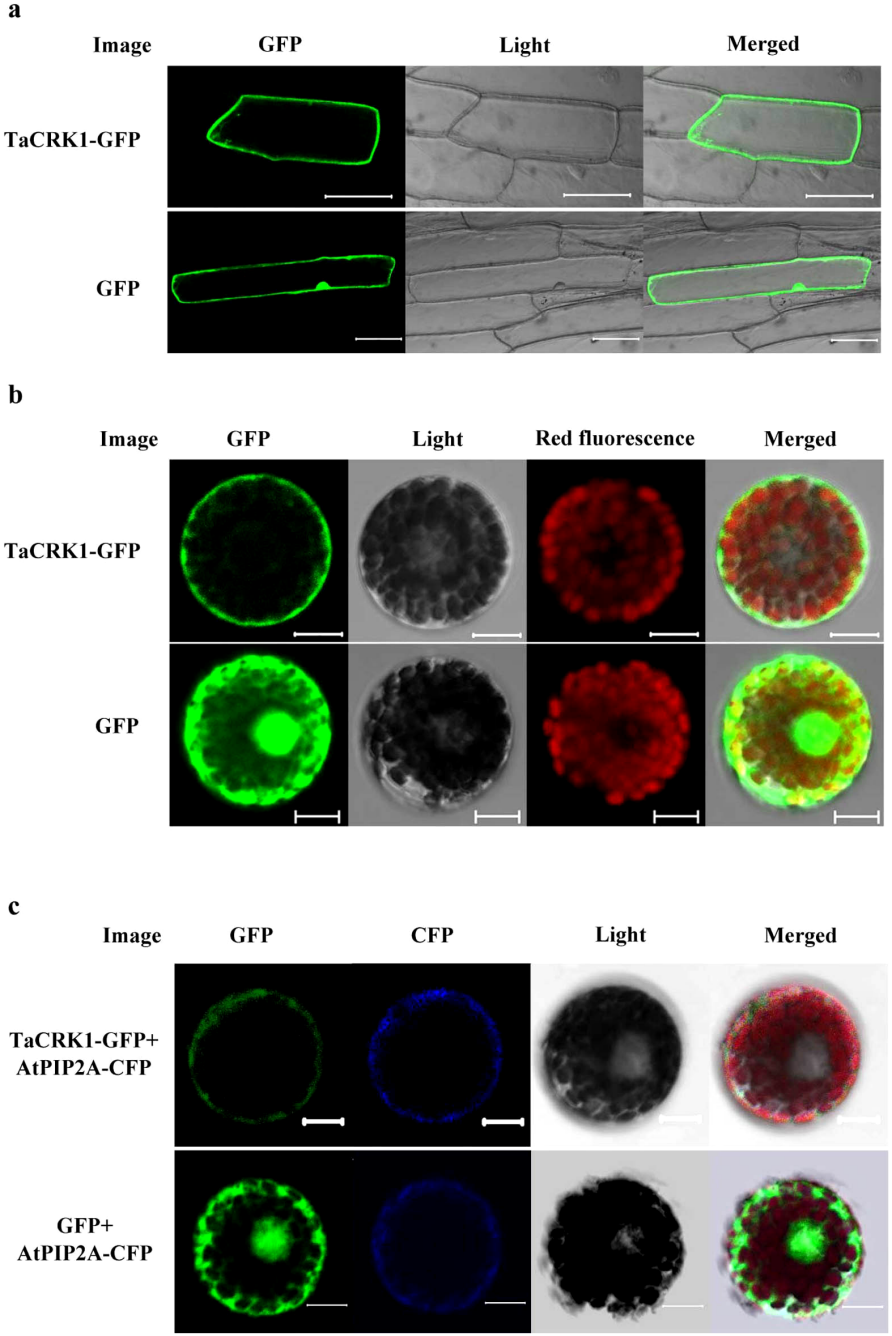
Plasma membrane localization of TaCRK1-GFP fusion protein. (a) Subcellular localization of the fused p35:TaCRK1-GFP in onion epidermal cells. The p35:TaCRK1-GFP and p35:GFP constructs were separately introduced into onion epidermal cells by bombardment and expressed under control of CaMV 35S promoter. Bars = 100 μM. (b) Subcellular localization of the fused p35:TaCRK1-GFP in wheat protoplasts. The p35:TaCRK1-GFP and p35:GFP constructs were separately introduced and expressed transiently in wheat protoplasts. Bars = 10 μM. (c) Co-localization of the fused p35:TaCRK1-GFP and CFP-labeled plasma membrane marker AtPIP2A in wheat protoplasts. The TaCRK1-GFP and AtPIP2A were co-transformed into wheat protoplasts and co-expressed under control of CaMV35S promoter. Bars = 10 μM.

To study the subcellular location of TaCRK1, p35S:TaCRK1-green fluorescence protein (GFP) fusion expressing vector was generated and introduced into onion epidermal cells or wheat protoplasts, using p35S:GFP construct as the control. Confocal microscopic observations showed that the transient expression of p35S:TaCRK1-GFP localized to cell periphery both in the onion epidermal cells and in the wheat protoplasts, whereas GFP protein alone was distributed in the entire cytoplasm and nucleus (Fig. 4a–b), suggesting that TaCRK1 seems to be a plasma membrane protein *in planta*. To further confirm this localization, a cyan fluorescent protein (CFP)-labeled plasma membrane marker AtPIP2A^28^ construct and the TaCRK1-GFP construct were co-transformed into wheat protoplasts and then co-expressed. AtPIP2A was an *Arabidopsis* plasma membrane intrinsic protein, which is often used as a plasma membrane targeted marker. The co-expression of AtPIP2A-CFP together with other plant protein was used to study the subcellular localization of the plant proteins, including barley (*Hordeum vulgare* L.)^29^, *Medicago truncatula*^30^, and *Oncidium* Gower Ramsey^31^. Here, the AtPIP2A-CFP fusion protein localized to plasma membrane in wheat protoplast from 15 h to 18 h after transformation (Fig. 4c), similar to the plasma membrane localization pattern of AtPIP2A in *Arabidopsis* and *Medicago truncatula*. TaCRK1-GFP also exhibited a plasma membrane localization pattern in wheat protoplast from 15 h to 18 h after transformation (Fig. 4c). The merging images obtained from the GFP and CFP channels showed that the TaCRK1-GFP and AtPIP2A-CFP fluorescence proteins co-localized to the plasma membrane (Fig. 4c), suggesting that TaCRK1 protein was likely to be a the plasma membrane protein in wheat. These results were consistent with those of RLKs that typically function in the cellular membrane.

### Expression of *TaCRK1* was induced by exogenous ABA stimuli

**Figure 5:**
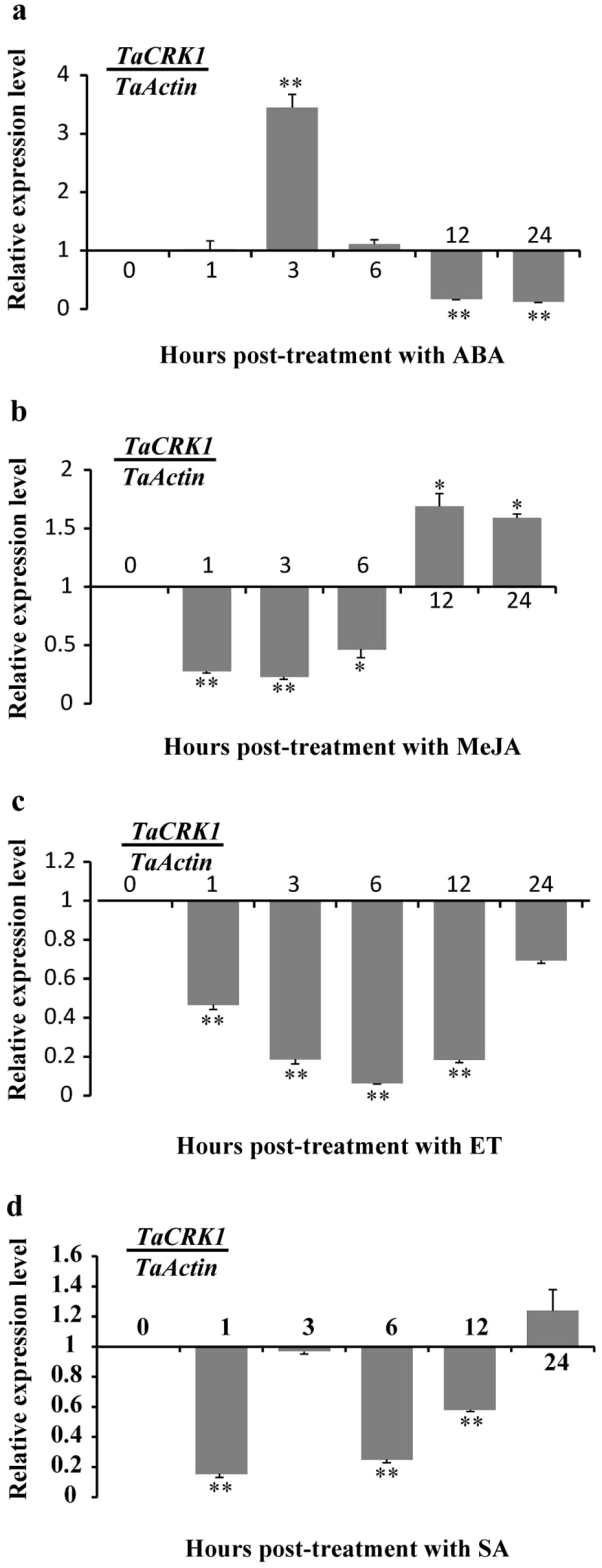
Transcriptional patterns of *TaCRK1* in wheat before (0 h) and after treatments by exogenous phytohormones ABA (a), MeJA (b), ET (c) and SA (d) for 1, 3, 6, 12 and 24 h. Relative expression of *TaCRK1* was relative to the control (0 hpt). Three biological replicates for each time point were averaged with standard error of mean indicated. Asterisks indicate statistically significant variation calculated using Student's t-test (*P<0.05, **P<0.01).

Certain RLKs have implicated in hormone signal transduction. To determine if the transcript of *TaCRK1* is induced by phytohormones including abscisic acid (ABA), jasmonic acid (JA), ethylene (ET), and salicylic acid (SA), qRT-PCR was used to investigate the transcriptional patterns of *TaCRK1* in *R. cerealis*-resistant wheat CI12633 across a time course taken at 0, 1, 3, 6, 12 and 24 h after treatment with the exogenous hormones. As shown in Fig. 5a, the transcriptional level of *TaCRK1* increased at 1–6 hours post-treatment (hpt), reached a peak at 3 hpt (more than three-fold over that of 0 hpt) and then decreased at 12–24 hpt. Upon MeJA treatment, the expression of *TaCRK1* decreased at 1–6 hpt, but slightly increased at 12–24 hpt (Fig. 5b). Upon ET treatment, the transcriptional level of *TaCRK1* decreased from 1 to 24 hpt (Fig. 5c). Upon SA treatment, the expression of *TaCRK1* decreased from 1 to 12 hpt, but at 24 hpt it increased close to non-treated level (Fig. 5d). To understand the putative molecular basis of *TaCRK1* in these responses, we analyzed *cis*-elements in the 1899-bp upstream of the start codon of *TaCRK1*. Analysis showed that the promoter contained six ABA-responsive elements (ABRE) (core sequence, PyACGTGG/TC)^32^, among which the box between −1753 and −1746 (CACGTGTC, in *trans* orientation) is a typical ABRE (shown in Supplementary Table 3 and Supplementary Fig. 2). These results suggested that *TaCRK1* may be involved in the ABA signaling pathway.

### Down-regulation of *TaCRK1* transcript did not obviously impair *R. cerealis* resistance

**Figure 6:**
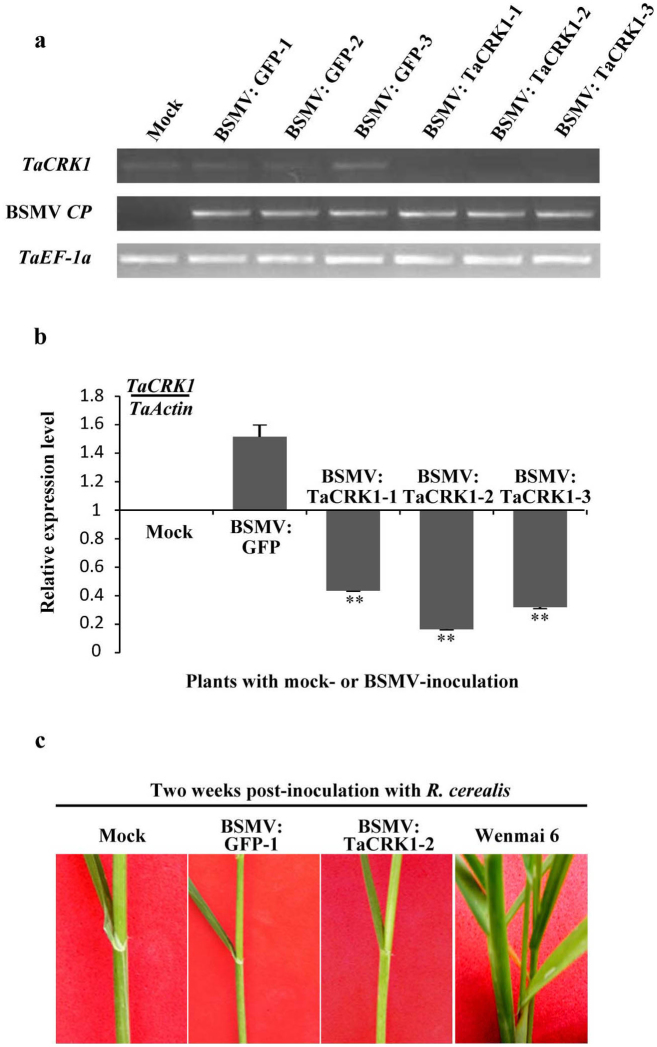
Effect of *TaCRK1* silencing on the resistance response of CI12633 to necrotrophic pathogen *Rhizoctonia cerealis*. (a) Relative transcript levels of *TaCRK1* and BSMV *CP* genes in the 4^th^ leaves of the mock plants or those infected by BSMV:GFP and BSMV:TaCRK1 as evaluated by semi-quantitative RT-PCR using gene-specific primers. Amplification of the wheat *TaEF-1a* gene served as internal control. (b) Relative transcript levels of *TaCRK1* in the 4^th^ leaves of the mock plants or those infected by BSMV:GFP and BSMV:TaCRK1 as evaluated by qRT-PCR. Relative expression of *TaCRK1* indicated the changing fold of the gene transcript over plants with mock-treatment. Three biological replicates were averaged with standard error of mean indicated. Asterisks indicate statistically significant variation calculated using Student's t-test (**P<0.01). (c) Response of the 4^th^ sheaths of the mock and BSMV virus-inoculated CI12633 and positive control Wenmai 6 to *R. cerealis*. The photographs were taken 2 weeks after *R. cerealis* inoculation. Gels in Fig. 6 were cropped and full-length gels are presented in the Supplementary Fig. 3, 4 and 5.

To investigate whether *TaCRK1* plays an important role in wheat resistance to *R. cerealis*, *TaCRK1* transcript level was knocked down in resistant wheat CI12633 using a virus-induced gene silencing (VIGS) technique. VIGS was developed with barley stripe mosaic virus (BSMV) and demonstrated to be an effective reverse genetics tool for investigating the functions of some genes in barley and wheat^33^,^34^,^35^,^36^,^37^. The RNAγ cDNA clone of BSMV can be manipulated to accommodate the transcription of non-viral sequences in infected barley or wheat plants^34^. In this study, a 298-bp fragment comprising the 3′ end of the ORF and part of the 3′ UTR sequence was inserted in an antisense orientation into *Nhe*I restriction site of the RNAγ to generate the BSMV:TaCRK1 construct. Semi-quantitative RT-PCR analyses showed that the transcript of the BSMV *CP* gene was detected in both BSMV:GFP- and BSMV: TaCRK1-inoculated CI12633 plants, but not in the mock (buffer-inoculated) plants (Fig. 6a). As expected, the *TaCRK1* transcript level was substantially reduced in CI12633 plants infected by BSMV: TaCRK1 (Fig. 6a–b), proving that the *TaCRK1* expression was suppressed in these CI12633 plants infected by BSMV:TaCRK1.

At the tillering stage, the 4^th^ sheaths in the mock CI12633 plants and those infected with the recombinant BSMV viruses were further inoculated with mycelia of *R. cerealis*. As positive control for successful *R. cerealis* inoculation, the 4^th^ sheath of Wenmai 6 was also infected with mycelia of *R. cerealis*. At 2 weeks post inoculation with *R. cerealis*, a dark-brown margin (an early symptom of sharp eyespot disease) was present at the 4^th^ sheaths of susceptible Wenmai 6 but absent in BSMV:TaCRK1-inoculated, BSMV:GFP-inoculated, and mock CI12633 plants (Fig. 6c). Furthermore, until the mature stage, no sharp eyespot symptom was observed at 4^th^ sheaths and stems of BSMV:TaCRK1-inoculated, BSMV:GFP-inoculated, and mock CI12633 plants, but the obvious symptoms were present at 4^th^ sheaths and stems of Wenmai 6 plants. These results suggested that *TaCRK1* silencing did not directly compromise the wheat resistance to *R. cerealis* in CI12633.

## Discussion

Plant receptor protein kinases, representing the main plasma membrane receptors, play important roles in perceiving extracellular signals and triggering rapid resistance responses^38^. In this study, we isolated a wheat CRK gene, *TaCRK1*, from *R. cerealis*-resistant wheat CI12633, based on a sequence differentially expressed between resistant wheat CI12633 and susceptible wheat Wenmai 6. *TaCRK1* transcript was rapidly induced by *R. cerealis* infection in resistant line CI12633 and was more than 2-fold higher in CI12633 than in Wenmai 6, suggesting that *TaCRK1* might be involved in wheat defense responses to *R. cerealis*. The deduced protein possesses a signal peptide domain, two extracellular DUF26 domains (each containing one copy of CRR motif), a transmembrane domain, and a kinase catalytic domain including 11 kinase subdomains. Phylogenetic analysis revealed that TaCRK1, together with BdCRK, ZmCRK, OsCRK, and AtCRK13 fell into the CRK clade of RLK proteins. Thus, TaCRK1 is a novel member of the CRK subgroup of RLK family in wheat. Certain CRK proteins from *Arabidopsis thaliana* have been implicated in defense responses; for instance, overexpression of *Arabidopsis AtCRK5* was correlated with enhanced leaf growth and displayed enhanced resistance to bacterial pathogen *Pseudomonas syringae* through induction of expression of *pathogenesis-related 1* (*PR1*) gene^15^.

Cells of eukaryotic organisms are organized into a large number of compartments to carry out a large number of biochemical functions. According to the intracellular localization of an uncharacterized protein, the likely functions of this protein can be infered^39^. Onion epidermal cells and wheat protoplasts were used in this study to investigate the subcellular localization of TaCRK1. In the onion cells, p35S:TaCRK1-GFP localized to the cell periphery; in wheat protoplasts, TaCRK1-GFP appeared to localize to the plasma membrane; whereas p35S:GFP localized to the entire cytoplasm and nucleus, suggesting that TaCRK1 protein was likely to targete to the plasma membrane in wheat and onion plants. AtPIP2A is a plasma membrane aquaporin and belongs to the plasma membrane intrinsic protein family^28^. Recent studies showed that AtPIP2A was an excellent cell plasma membrane marker in *Arabidopsis*, barley, *Medicago truncatula*, and *Oncidium* Gower Ramsey^29^,^30^,^31^,^39^. For instance, a HvPHT1;6:GFP was transiently co-expressed with either the plasma membrane targeted marker, AtPIP2A:mCherry, or the vacuolar membrane marker, gTIP:mCherry. The green fluorescence of HvPHT1;6:GFP co-localized with the red fluorescence of the plasma membrane marker AtPIP2A:mCherry, separated from that of the red fluorescence of the vacuolar marker gTIP:mCherry^29^. In this study, AtPIP2A-CFP protein exhibited to a plasma membrane localization pattern in wheat protoplasts, and fluorescence from TaCRK1-GFP and AtPIP2A-CFP seemed to be co-localized to the plasma membrane. Because there is no reported research that AtPIP2A reliably localizes to the plasma membrane in wheat protoplasts, the localization results of TaCRK1-GFP need further to be proved.

Many RLKs have been shown to be involved in hormonal signal transduction^18^,^40^,^41^. For instance, upregulation of *AtCRK13* in *Arabidopsis* led to hypersensitive response-associated cell death and induced defense against pathogens by causing increased accumulation of salicylic acid^14^. In plants, the ABA pathway has been implicated in regulation of plant development and response to biotic and abiotic stresses^42^,^43^,^44^. A receptor-like kinase in *Arabidopsi, GUARD CELL HYDROGEN PEROXIDE-RESISTANT1* (*GHR1*), was shown to be a critical component in ABA and H_2_O_2_ signaling pathways and to be involved in stomatal movement^41^. The tomato ABA-inducible MYB transcript factor AIMI (abscisic acid-induced myb1) was suggested to function in ABA sensitivity, abiotic stress tolerance, and basal resistance against *Botrytis cinerea* in tomato^45^. Most ABA-inducible genes contain a conserved, ABA responsive, *cis*-acting element, designated as ABRE (core sequence, PyACGTGG/TC), in their promoter regions^46^. It was found that the expression of ABA responsive gene requires multiple ABREs or the combination of an ABRE with a coupling element (CE) as a functional promoter^47^,^48^. In this study, qRT-PCR analyses revealed that *TaCRK1* in resistant wheat CI12633 could be rapidly induced by exogenous ABA treatment. The 1899-bp promoter of *TaCRK1* contained one ABRE and five ABRE-like boxes, which may partially contribute to the response of *TaCRK1* to ABA stimuli. In addition, the transcript level of *TaCRK1* was reduced by MeJA and ET treatments. No JA- or ethylene- or SA-responsive element was detected in the promoter of *TaCRK1*, suggesting that *TaCRK1* indirectly regulated by MeJA or ET or SA. These results suggested that *TaCRK1* might be involved in other responses regulated by ABA signaling pathway, which will be further studied in the future.

VIGS is an efficient tool for rapidly analyzing plant gene functions. In this study, the VIGS approach was utilized to investigate the function of *TaCRK1* in wheat defense response to *R. cerealis*. Although the *TaCRK1* transcript level was reduced in resistant CI12633 plants infected by BSMV:TaCRK1, down-regulation of *TaCRK1* in CI12633 did not obviously impair wheat resistance to *R. cerealis*. Plant immunity is a complex network in which some components and network sectors interact with each other in complex ways. The function of a sector of the network can be compensated by some other sectors; consequently, functional identification of these sectors only by knocking out each of the sectors is difficult^49^. For example, *BRL1* is functionally redundant with *BRI1* in regulating *Arabidopsis* brassinosteroid signaling. The brl1-1 mutant plants did not have obvious phenotypes, but bri1-5 brl1-1 double mutants showed enhanced defective leaf phenotypes compared with the bri1-5 single mutant^50^. In this study, reducing *TaCRK1* expression did not compromise CI12633 resistance to *R. cerealis*. The reason might be that *TaCRK1* is not the major gene controlling wheat defense response to *R. cerealis* or that *TaCRK1* is functionally redundant with some other genes. Responses of *TaCRK1* in other environmental stresses will be further investigated in the future.

In summary, *TaCRK1*, the first DUF26-CRK gene isolated from wheat, was identified via RNA-seq and characterized. It undergoes significantly higher expression levels in resistant wheat CI12633 following *R. cerealis* infection and exogenous ABA stimuli. *TaCRK1* encodes a cysteine-rich receptor-like protein kinase TaCRK1. The TaCRK1 protein localizes to the plasma membranes in wheat protoplasts and in onion epidermal cells. Our results give new insights into the CRK subgroup of the RLK family in plant species, and may pave the way to further study of the functions of CRKs in wheat.

## Methods

### Plant and fungal materials and treatments

Seven wheat (*Triticum aestivum* L.) lines/cultivars, CI12633, Shanhongmai, Navit14, Shannong0431, Xifeng, Wenmai 6, and Yangmai 158, exhibit different levels of resistance and susceptibility to *R. cerealis*. The pathogenic fungus *Rhizoctonia cerealis* isolate R0301 was provided by Profs. Huaigu Chen and Shibing Cai at Jiangsu Academy of Agricultural Sciences, China.

Wheat plants were grown in a 16 h light/8 h dark (22°C/12°C) regime. At the tillering stage, each 2^nd^ base sheath of wheat plants was inoculated with small toothpick fragments harboring the well-developed mycelia of *R. cerealis*. Mock (control) plants were inoculated with small toothpick fragments soaked in liquid potato dextrose agar (PDA). Inoculated plants were grown at 90% relative humidity for 4 days. The inoculated stems were sampled at 4 days post inoculation (dpi), quickly frozen in liquid nitrogen, and stored at −80°C prior to extraction of total RNA.

The seedlings of wheat line CI12633 at three-leaf stage were treated with phytohormones, such as 1.0 mM SA, 0.1 mM methyl jasmonate (MeJA, JA analog), ethylene (ET) released from 0.2 mM ethephon, and 0.2 mM abscisic acid (ABA) following the method described by Zhang et al^51^. At 0, 1, 3, 6, 12 and 24 h after treatments with SA, ethylene, MeJA, or ABA, the leaves were collected for RNA extraction.

### RNA extraction and cDNA synthesis

Total RNA was extracted using TRIzol reagent (Qiagen, China) according to the manufacturer's instructions. DNase I treatment was applied to remove contaminated genomic DNA. The first-strand cDNA was synthesized using 2 μg purified RNA, AMV reverse transcriptase, and oligo (dT_15_) primers (TAKaRa, Japan) according to the manual.

### Cloning and sequence analysis of *TaCRK1*

The sequence of the 3′ un-translated region (UTR) was amplified from cDNA of the CI12633 stems challenged with *R. cerealis* for 4 days using 3′-Full RACE Core Set Ver.2.0 (TaKaRa, Japan) based on the wheat cDNA clone AK330939. Then, two pairs of primers (*TaCRK1*-1^st^-F/*TaCRK1*-1^st^-R and *TaCRK1*-2^nd^-F/*TaCRK1*-2^nd^-R, Supplementary Table 2) were designed and used to amplify the full open reading frame (ORF) sequence of *TaCRK1* from the cDNA of the CI12633 through two rounds of nested RT-PCR. The resulting PCR products were cloned to the pMD-18T Vector (TaKaRa, Japan) to form the positive clones. At least five positive clones were sequenced with an ABI PRISM 3130XL Genetic analyzer (Applied Biosystems, Foster City, CA). cDNA sequence data were analyzed using BLAST (http://blast.ncbi.nlm.nih.gov/Blast.cgi) and ORF Finder (http://www.ncbi.nlm.nih.gov/gorf/). The deduced protein sequence analyses were performed using the Compute pI/MW tool (http://web.expasy.org/compute_pi/) for computation of the theoretical iso-electric point and protein molecular weight, InterPro-Scan (http://www.ebi.ac.uk/interpro/) and Smart software (http://smart.embl-heidelberg.de/smart/set_mode.cgi?GENOMIC=1) for prediction of the conserved domains and motifs, DNAMAN software for sequence alignment, and MEGA 5.0 software for constructing a phylogenetic tree. The upstream region (1899 bp) to start codon was analyzed for detection of ABA responsive elements using the plant *cis*-acting regulatory DNA elements (PLACE) database^52^ (http://www.dna.affrc.go.jp/PLACE/).

### Subcelluar localization of TaCRK1

The coding region of *TaCRK1* without the stop codon was amplified using gene-specific primers with *Pst*I and *Xba*I restriction sites (5′-GTCCTGCAGATGGCCAAACCCCACCGC-3′, with underline denoting the *Pst*I site; and 5′-GCTCTAGATCTTGGCGAAAGCTCCGT-3′, with underline denoting the *Xba*I site) and was subcloned in-frame to the 5′-terminus of the GFP coding sequence in p35S:GFP vector (Dr. Daowen Wang, Chinese Academy of Sciences), generating the TaCRK1-GFP fusion construct p35S:TaCRK1-GFP.

The resulting p35S:TaCRK1-GFP or p35S:GFP alone construct was separately bombarded into white onion epidermal cells following Zhang et al^26^. The TaCRK1-GFP fusion or GFP alone construct was separately introduced into wheat protoplasts via the PEG-mediated transfection method following Yoo et al^53^, using CFP-labeled plasma membrane marker AtPIP2A in vector CD_3_-1002^28^ as control. For expression of the introduced GFP proteins, the transformed wheat protoplasts or onion cells were incubated at 25°C for 15 h. The GFP and CFP signals were then observed and photographed using a Confocal Laser Scanning Microscopy (Zeiss LSM 700, Germany) with a Fluar 10X/0.50 M27 objective lens and SP640 filter.

### Functional analysis of *TaCRK1* through virus-induced gene silencing

To generate the BSMV:TaCRK1 construct, a 298-bp sequence of *TaCRK1* (from 1913 to 2211 nucleotides in the *TaCRK1* cDNA sequence) was amplified from CI12633 stem cDNA with the primers (5′-GACGCTAGCTCCCTTCTCTGTCCAGGC-3′ and 5′-CGCGCTAGCTAGCATCTTAGCAGTTCTAC-3′; underlined sections are the *Nhe*I restriction sites). Then the fragment was inserted in an antisense orientation into *Nhe*I restriction site of the RNAγ, resulting in the recombinant construct RNAγ:TaCRK1-as. Following a previously described protocol^42^, the tripartite cDNA chains of BMSV: TaCRK1-as, or the control virus BMSV:GFP genome, were separately transcribed into RNAs and then mixed to infect CI12633 plants at the two-leaf stage. At the same time, CI12633 plants were inoculated only with the buffer containing no virus; hereafter, these plants are called mock treatments. The 4^th^ leaves of the inoculated seedlings were collected to monitor BSMV infection based on the transcripts of BSMV coat protein (*CP*) gene using BSMV-*CP*-F/BSMV-*CP*-R primers and to evaluate the transcript changes of *TaCRK1* with *TaCRK1*-Q-F/*TaCRK1*-Q-R primers (Supplementary Table 2). For *R. cerealis* inoculation, the fungus was cultured on potato dextrose agar at 25°C for 10 days, then 1-cm^2^ plugs from the edge of *R. cerealis* colonies were placed into liquid PDA medium and cultured at 25°C for 2 weeks to develop mycelia. At the tillering stage, the 4^th^ base sheath of wheat plants was inoculated with 15 μl culture of *R. cerealis*. Inoculated plants were grown at 90% relative humidity for 4 days. Sharp eyespot symptoms were observed at 14 days and 40 days after the fungal inoculation, when sharp eyespot symptoms were present at the infected sheaths and stems, respectively, of susceptible Wenmai 6.

### RT-PCR and Real-time quantitative RT-PCR (qRT-PCR) analysis

RT-PCR was performed with the following thermal profile: initial denaturation at 94°C for 5 min; followed by 30 cycles of 30 s at 94°C, 30 s at 60°C, and 30 s at 72°C; and final extension at 72°C for 5 min. The PCR products were detected on 1.5% agarose gel. In all the semi-quantitative RT-PCR experiments, wheat *elongation factor 1 alpha-subunit* gene (*TaEF-1a*) was used to normalize the cDNA contents among various samples.

qRT-PCR was performed using SYBR Green I Master Mix (TaKaRa, Japan) in a volume of 25 μl on an ABI 7300 RT-PCR system (Applied Biosystems). Reactions were set up with the following thermal profile: 95°C for 5 min, followed by 41 cycles of 95°C for 15 s and 60°C for 31s, and completed with a melting curve analysis program. All qRT-PCR reactions were repeated three times. The relative expression of the gene *TaCRK1* was calculated with the 2^−ΔΔCT^ method^54^, where the wheat *TaActin* gene was used to normalize amounts of cDNAs among the samples.

The sequences of primers were listed in Supplementary Table 2.

## Author Contributions

Z.Z. and K.Y. designed the research, interpreted the data, and wrote the paper. K.Y. performed the cloning, sequencing, subcellular localization, VIGS and functional assays. W.R. identified the wheat cDNA clone AK330939. L.Q. performed qRT-PCR analysis. J.L. modified the manuscript. X.W. prepared the recombinant virus construct in VIGS and inoculation.

## Supplementary Material

Supplementary Information Supplementary information
