# Supplementary material for: Isolation and characterization of a novel wheat cysteine-rich receptor-like kinase gene induced by Rhizoctonia cerealis
Source: Sci Rep. 2013 Oct 23;3:3021. doi: 10.1038/srep03021 (PMC3805973; doi:10.1038/srep03021)

Isolation and characterization of a novel wheat cysteine-rich receptor-like kinase gene induced by *Rhizoctonia cerealis*

Kun Yang, Wei Rong, Lin Qi, Jiarui Li, Xuening Wei, Zengyan Zhang

Supplementary Table 1

***Rhizoctonia cerealis* reponses of seven wheat lines/cultivars**

Wheat lines/cultivars	Average disease index (%)		Response degree
	Filling stage	Mature stage	
CI12633	26.67	30.17	R
Shanhongmai	20	31.81	R
Navit14	24.55	38.31	MR
Shannong0431	30	36.67	MR
Xifeng	27.37	35.27	MR
Yangmai 158	34.48	52.44	MS
Wenmai 6	46.92	75.22	S

R, resistant; MR, moderately resistant; MS, moderately susceptible; S, susceptible.

Supplementary Table 2

Sequences of the gene-specific primers used in the study

Primer name	Gene Accession	Sequences of gene-specific primer	Usage
TaCRK1-1st-F	KC818618	5’-GTTTACCGAGGCAGTGAGC-3’	Amplification of full-length cDNA of *TaCRK1*
TaCRK1-1st-R		5’-ATACAGCACTACAGCAGGGC-3’	
TaCRK1-2nd-F		5’-CACCGCTGACGGATTG-3’	
TaCRK1-2nd-R		5’-AGATACTATCCCGTAAGAAAACT-3’	
TaCRK1-3’-F_1		5’-CACCGAGACCTCAAAGCG-3’	3’ RACE for *TaCRK1*
TaCRK1-3’-F_2		5’-AGATTTCTGACTTCGGCTTAG-3’	
TaCRK1-Q-F		5'-CGACCAGGTGCTCAAGTGC-3'	qRT-PCR for *TaCRK1* transcript
TaCRK1-Q-R		5'-TCAGGACTCATCTTGGCGAA-3'	
BSMV-*CP*-F	JF803284	5’-TGACTGCTAAGGGTGGAGGA-3’	Detection of BSMV virus
BSMV-*CP*-R		5’-CGGTTGAACATCACGAAGAGT-3’	
TaEF-1a-F	M90077	5’-CAGATTGGCAACGGCTACG-3’	Internal control for semi-qRT-PCR
TaEF-1a-R		5’-CGGACAGCAAAACGACCAAG-3	
TaActin-F	BE425627	5’-CACTGGAATGGTCAAGGCTG-3’	Internal control for qRT-PCR
TaActin-R		5’-CTCCATGTCATCCCAGTTG-3’	

Supplementary Table 3

Motifs in upstream region to the start codon detected using PLACE database

Motif	Signal sequence	Location
-10PEHVPSBD	TATTCT	-490
ABRELATERD1	ACGTG	-1751,-286,-1858,-1752,-1593
ABRERATCAL	MACGYGB	-1752,-1753,-1859,-1126,-868
ACGTABOX	TACGTA	-346
ACGTABREMOTIFA2OSEM	ACGTGKC	-1754,-1857,-1751,-1592,-1387,-1259,-1214,-391,-345,-297,-286,-1857,-1751,-1592,-1387,-1259,-1214,-391,-345,-297,-286
AMYBOX1	TAACARA	-824,-451
ANAERO1CONSENSUS	AAACAAA	-1775,-1654,-520,-132,-126,-636,-1132,-769
ARR1AT	NGATT	-1886,-1468,-1762,-698,-548,-82,-435,-149,-1700,-1398,-709,-291
ASF1MOTIFCAMV	TGACG	-1312,-1261,-1216,-678,-87,-497,-390,-296,-260
BIHD1OS	TGTCA	-421,-1768,-1707
BOXCPSAS1	CTCCCAC	-1511
BOXLCOREDCPAL	ACCWWCC	-1901,-23,-360
BP5OSWX	CAACGTG	-288,-1858
CAATBOX1	CAAT	-1701,-350,-546,-80
CACGTGMOTIF	CACGTG	-1752
CACTFTPPCA1	YACT	-1901,-1515,-1415,-1487,-728,-462,-431,-365,-1843,-1840,-1822,-1745,-1709,-1574,-1552,-1489,-1402,-1189,-749,-713,-703,-617,-603,-538,-423,-373,-370,-367,-243,-100
CANBNNAPA	CNAACAC	-1519
CAREOSREP1	CAACTC	-1584,-776
CATATGGMSAUR	CATATG	-1676
CBFHV	RYCGAC	-182,-178,-686,-1440,-1326,-1221
CCAATBOX1	CCAAT	-1702,-546
CGACGOSAMY3	CGACG	-1495,-1240,-1105,-684,-1386,-1381,-1291,-1168,-806,-786,-562,-33
CGCGBOXAT	VCGCGB	-997,-576,-1279,-1273,-1267,-1096,-1070,-1126,-1054,-1036,-962,-883,-868,-997,-576,-1279,-1273,-1267,-1096,-1070,-1126,-1054,-1036,-962,-883,-868
CIACADIANLELHC	CAANNNNATC	-715
CURECORECR	GTAC	-1488,-1389,-1377,-742,-729,-616,-443,-377,-366,-117,-1488,-1389,-1377,-742,-729,-616,-443,-377,-366,-117
DOFCOREZM	AAAG	-1868,-1689,-622,-310,-1898,-1894,-1656,-513,-225,-153
DPBFCOREDCDC3	ACACNNG	-1753,-174,-1752
DRE2COREZMRAB17	ACCGAC	-182,-178,-1221
DRECRTCOREAT	RCCGAC	-182,-178,-686,-1440,-1326,-1221

EBOXBNNAPA	CANNTG	-1772,-1752,-1711,-1676,-634,-557,-540,-522,-257,-231,-198,-1772,-1752,-1711,-1676,-634,-557,-540,-522,-257,-231,-198
EECCRCAH1	GANTTNC	-1885,-1584,-697,-148,-1645,-1401,-1204,-712
ELRECOREPCRP1	TTGACC	-527,-543,-216
GADOWNAT	ACGTGTC	-1754
GARE1OSREP1	TAACAGA	-824
GATABOX	GATA	-483,-448,-187
GCCCORE	GCCGCC	-1628,-1436,-1283,-975,-791,-1601,-1335,-1296,-1113,-1110,-1026,-918,-840
GT1CONSENSUS	GRWAAW	-1479,-1884,-696,-505,-147,-695,-111
GTGANTG10	GTGA	-1819,-1708,-1217,-833,-602,-588,-537,-454,-426,-284,-99,-1659,-1640
HEXAMERATH4	CCGTCG	-787
HEXMOTIFTAH3H4	ACGTCA	-391,-297,-1261,-1216
INRNTPSADB	YTCANTYY	-605,-1852,-1605,-1177
LTREATLTI78	ACCGACA	-178,-1222
LTRECOREATCOR15	CCGAC	-1496,-685,-205,-181,-177,-1440,-1346,-1326,-1221
MYB2CONSENSUSAT	YAACKG	-1250
MYBCORE	CNGTTR	-1250,-1226,-1148,-654,-573,-338,-318,-824
MYBCOREATCYCB1	AACGG	-1250
MYBPLANT	MACCWAMC	-571
MYBPZM	CCWACC	-946
MYBST1	GGATA	-449
MYCATERD1	CATGTG	-557
MYCCONSENSUSAT	CANNTG	-1772,-1752,-1711,-1676,-634,-557,-540,-522,-257,-231,-198,-1772,-1752,-1711,-1676,-634,-557,-540,-522,-257,-231,-198
NODCON2GM	CTCTT	-1664,-1010
OSE2ROOTNODULE	CTCTT	-1664,-1010
PALBOXAPC	CCGTCC	-10
POLASIG1	AATAAA	-385
POLLEN1LELAT52	AGAAA	-1646,-406,-395,-312,-301,-1897,-1893,-1882,-1792,-516,-503
PRECONSCRHSP70A	SCGAYNRNNNNNNNNNNNNNNNHD	-181,-1240,-270
PYRIMIDINEBOXOSRAMY1A	CCTTTT	-154
RAV1AAT	CAACA	-318,-234,-1255,-1225,-955,-653,-198
RHERPATEXPA7	KCACGW	-1859,-1594,-286,-590
SEF4MOTIFGM7S	RTTTTTR	-129,-123
SORLIP1AT	GCCAC	-898,-248,-95,-26,-1319,-1157,-566,-532
SORLIP5AT	GAGTGAG	-604

SURECOREATSULTR11	GAGAC	-1547
TGBOXATPIN2	AACGTGS000458	-287,-1858
TATABOX5	TTATTT	-386
TATCCAOSAMY	TATCCA	-450
TATCCAYMOTIFOSRAMY3D	TATCCAY	-451
TGACGTVMAMY	TGACGT	-1261,-1216,-391,-297
TRANSINITDICOTS	AMNAUGGC	-508
TRANSINITMONOCOTS	RMNAUGGC	-508,-77
UPRMOTIFIIAT	CCNNNNNNNNNNNNCCACG	-595
WBOXATNPR1	TTGAC	-1313,-527,-542,-420,-389,-295,-236,-215
WBOXHVISO1	TGACT	-1661,-237
WBOXNTERF3	TGACY	-526,-1661,-237,-543,-216
WRKY71OS	TGAC	-1768,-1707,-1312,-1261,-1216,-678,-526,-87,-1660,-542,-496,-420,-389,-295,-259,-236,-215

R=A/G; Y=C/T; K=G/T; M=A/C; W=A/T;S=G/C; V=A/C/G; B=G/T/C; H=T/C/A; D=A/T/G; N=A/G/C/T.

Supplementary Fig. 1 Comparison of predicted amino acid sequences of TaCRK1 and that of AK330939 using DNAMAN software. The substitutions, deletions, and insertions were marked by open boxes.

```
TaCRK1     MAKPHRCFSPYLAGVAATFLLPVLYAPLAAADDEPPPWPICGPYPPGGNYTPNSTYQANI   60
AK330939   MAKPHRCFSPYLAGVAATFLLPVLYAPLAAADDEPPPWPICGPYPPSGNYTHNSTYQANI   60

TaCRK1     DLLSATLPRNASLSPALYATGDVGDVPDIVYGQALCRGDVANASACEACVAAAFRGAQRA  120
AK330939   DLLSATLPRNASLSPALYATGDVGDVPDIVYGQALCRGDVANASTCEACVAAAFRGAQRA  120

TaCRK1     CPLYKDVIIFYDLCQLRFSNRNFLLDDDYIVTTYTLLRSRVVATPAFDAAVGLLLNATAD  180
AK330939   CPLYKDVIIFYDLCQLRFSNRNFLLDDDYIVTTYTLLRSRVVATPAFDAAVGLLVNATAD  180

TaCRK1     HAVEDSSRRFGTGEEGFGDRRNPKIYALTQCAPEKTADVCRSCLSVIIGQLPNSFRGRTG  240
AK330939   HAVEDSSRRFGTGEEGFGDRRNPKIYALTQCAPEKTADVCRSCLGVIIGQLPNSFRGRTG  240

TaCRK1     GGMFGVWCNFRYEVYPFFPGRPLVQLPQFVERPPASAPPVTGGEEKKRNSAGKVLAILMP  300
AK330939   GGMFGVWCNFRYEVYPFFSGRPLVQLPQFVQRPPASAPPVTGGEEKKRNSAGKVLAILMP  300

TaCRK1     TIAVILATAVVYIFCWRKRRPKEDAYLPSTSDDIQHIDSLLLDLATLRIATDDFDNSKMA  360
AK330939   TIAVILAIQVVYFFCWRKRRP.EEVYLPSTSDDIQHIDSLLLDLATLRIATDDFDNSKML  359

TaCRK1     GKGGFGMVYKGVLPDGEEIAVKSFGQTSRQGIGELKSELVLVAKLHHKNLVRLVGVCLEE  420
AK330939   GKGGFGMVYKGVLPDGEEIAVKRLGQTSRQGIGELKSELVLVAKLHHKNLVRLVGVCLEE  419

TaCRK1     QEKILVYEYMPNRSLDMILFDSEKNKELDWGKRFKIINGIARGLQYLHEDSQLKIVHRDL  480
AK330939   QEKILVYEYMPNRSLDMILFDSERNKELDWGKRFKIINGIARGLQYLHEDSQLKIVHRDL  479

TaCRK1     KASNVLLDVDYNPKISDFGLAKIFGGDQSEDVTRRIAGTYGYMAPEYAMRGQYSVKSDVF  540
AK330939   KASNILLDVDYNPKISDFGLAKIFGGDQSEDVTRRIAGTYGYMAPEYAMRGQYSVKSDVF  539

TaCRK1     SFGVLVLEIITGRRNSGSYNTEQDVDLLNLVWEHWTRGNVVELMDPSLSNHPPVDQVLKC  600
AK330939   SFGVLVLEIITGRRNSGSYNTEQDVDVLNLVWEHWTRGNVVELMDPSLSNHPPVDQVLKC  599

TaCRK1     IHVGLLCVQRKPASRPTMSSVNIMFSSHTVRLPSLSRPAFCIQEVSVSETSTAYSEAYPL  660
AK330939   IHVGLLCVQRKPASRPTMSSVNIMFSSHTVRLPSLSRPAFCIQEVSVSETSTAYSEAYPL  659

TaCRK1     TENSTVMSSNEVSITELSPR                                          680
AK330939   TENSTVMSSNqVSITELSPR                                          679
```

Supplementary Fig. 2 Analysis of nucleotide sequence of upstream region (1899 bp) to start codon. ABRE-like boxes were highlighted in gray and a typical ABRE element was marked by the open box.

GGCTTTCTTTCTGCCGATTTTCTCCTCCTCCAAAAGCAAACGCACGTTGAAATGAGAAGT	−1840
AGTGCAGCAACTATAGGTAGTGTGATGGCCAAGATCAACCCTGTCCTATTTCTTGTTCGT	−1780
TCTGCAAACAAATGACATGGATTTCAGACACGTGTAGTGCAGACTGCAGAGAAGTTGTAT	−1720
TCCACCATCCAAGTGACACCAATCAAGCACCAAAGAACAGCAACCATATGCGCCTGAAGA	−1660
GTCACTTTTGTTTCAGAAACTCACCTTCTTCGGCCGCCGCTGGCGCTGGCGCTGGTGCAG	−1600
GCGGCAGCACGTCCGGGAGTTGCAGCAGTGGGCGTCCGGAGAAGAAGGAGTAGGTCTCGA	−1540
ACCGGAAGTTGCATCGCACGCCGAACACTCTCCCACCCGGCTTCCCGACGAAGTACTGTG	−1480
GGGTAAACTTCTCGATTATGTCCCTGAGGCAGCTCCGGCAGTCGGCCGCCTCCATGTCCG	−1420
GCGTGCACTGCGCCAGCGAGTAAATCTTGGGGTACGTCGCGTCGTACCCCTCCTCCCCCG	−1360
TGCCGAACCGCCGGGTCGGGTCCCCGGCGGCATAGTCGGCGGTGGCGTTGACGAGCCGGC	−1300
CGGAGGCGGCGTCGAACGCCGCCGCGGCCGCGCCCGCGCTGACGTTGTTGCCGTTGAACG	−1240
CGACGAACCTGCCGCTGTTGTCGGTGACGTTGGCCAGGAAGTCCTGGCCGGAGTAGCGGA	−1180
GGATGCAGGGGTCGTCGAACATGGTGGCGTGCCGGTTGAAGGCGCAGAGCTGCTGCGCGT	−1120
TCCGGAAGGCGGCGGCGACGCAGGCCGCGCAGGAGGAGGCGTTGGTGTCGCCGCGGCAGA	−1060
GCGCGAGCGCGTAGACGGCGTCCGGCGCGGCGCCGGCGGCGCCCTTGGCGAAGAGCGCCG	−1000
GGGACGCGGAGGCGTTCCTGGGGAGGCCGCCGGCGAGGGCGCGTATGTTGGCCTGGTAGG	−940
CGCTGCCCTCCGTGTAGTTCCCGGCGGCGGAGTCGCAGAGCTGCCACGGGAGCGGCTGCG	−880
CGCCGGCGAGCGGCGCGTGGAGGAACGCGAGGAGGAGGAGGGCGGCGGTGAGGTGGTAAC	−820
AGAGAGCAAGGCTGCGTCGCATGCGCATGGCCGCCGTCGCCATGGAGTTGAGCTGCTGGG	−760
GGTGTCGCCGAGTAGATGTACAGCACGGGCGTACTGGTAGTTTCCAAGTAAATCGAAGTG	−700
TGGATTTTCCACGGCCGACGCTGACGAACGAACAGACCGATCGAACTGTTGCTGCCTTGG	−640
CGCAGCAGCTGATGTCGAAAGGAGTACGATGAGCAGAGTGAGGTCGTGGTCGTGAGCGAG	−580
CGGACGCGGTTAGGTGGCGTCGCACATGGTGGGATTGGTCAAGTGAGGTGGCTTGACCAT	−520
TTGTTTCTTTGGCCATTTTCTGCGTCAGGTATTCTAGATAGCTACGGCGTGCTGGACTAC	−460
TGGCGGTGATGGATAGGTACGGCCTGATTACTGGTGAGTGTCAAGATGAACAGAGAAAAC	−400
TTGGAGAAACGTCAAATAAACTGTACAGTAGTAGTACTAGGATGGTTCGCAATTACGTAT	−340
TCGGTTACAAATTCTTCTGATCAACAGAGAAAGCTTGAAGAAACGTCAAATCAACGTGAA	−280
ATAGTTGCTCGCCATTAGCCGTCAGATGGTCGCCACAGTAGCAGTCAACAAATGCTTTGC	−220
TCCGGTCAACCCCTCCGACCCCACCTGCTAATGATACACCGACCGACACCCGAACAACCC	−160
CTTCTCCTTTTGATTTCCTTCTTGTTTTTTGTTTTTGTTTTTGTACAGTTTACCGAGGCA	−100
GTGAGCCACCGCTGACGGGATTGCCATGGCCAAACCCCACCGCTGCTTCTCCCCGCACCT	−40
CGCCGGCGTCGCTGCCACCTTCCTCATCTCCGTCCTCCA	−1

Supplementary Fig. 3 Amplification of *TaCRK1* in the 4th leaves of the mock-inoculated seedlings or those infected by BSMV:GFP and BSMV:TaCRK1.

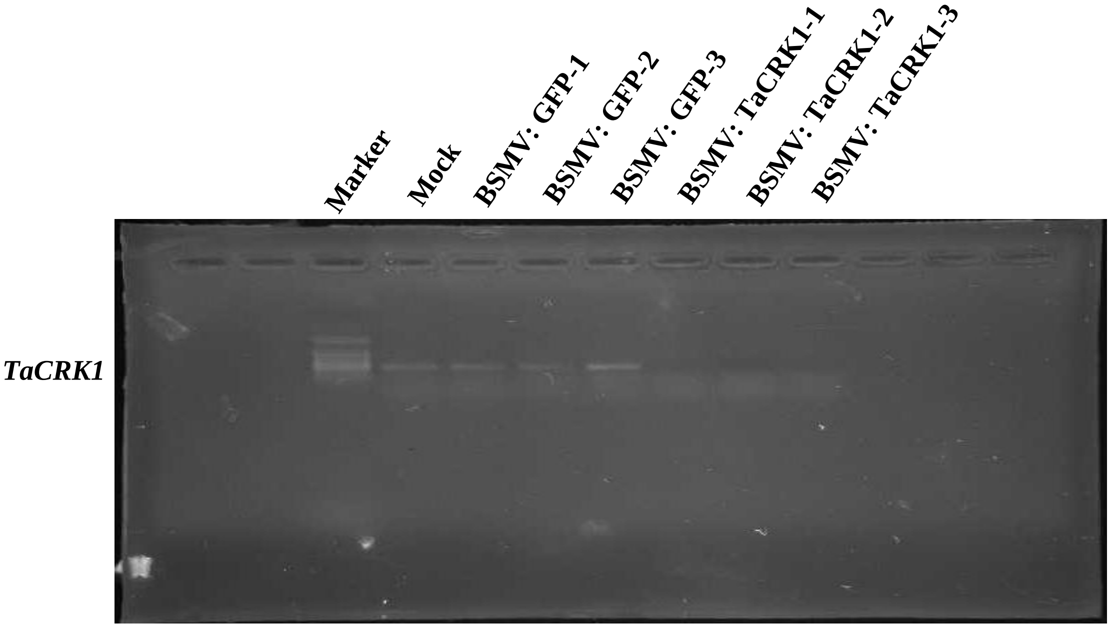

Supplementary Fig. 4 Amplification of BSMV *CP* genes in the 4th leaves of the mock-inoculated seedlings or those infected by BSMV:GFP and BSMV:TaCRK1.

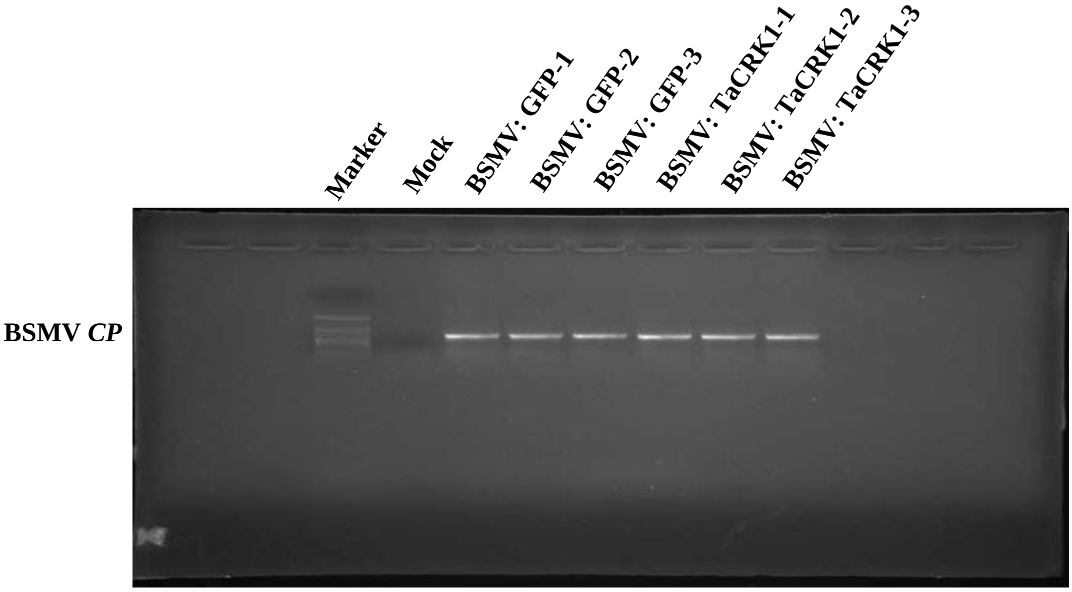

Supplementary Fig. 5 Amplification of *TaEF-1a* genes in the 4th leaves of the mock-inoculated seedlings or those infected by BSMV:GFP and BSMV:TaCRK1.

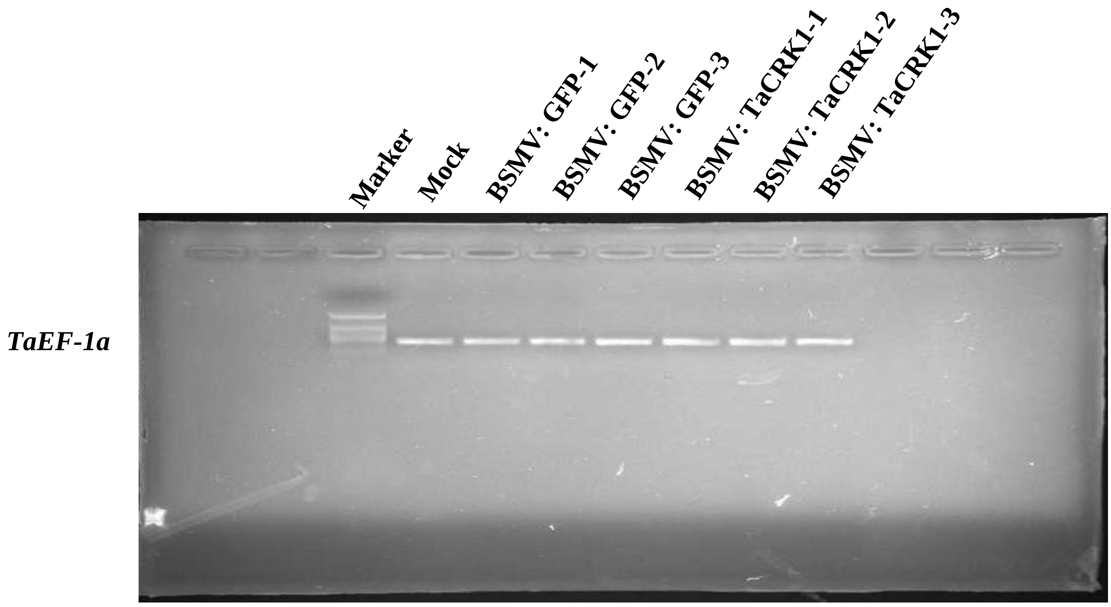

Supplement: Supplementary Information [file srep03021-s1.pdf]
